# Detection of human cytomegalovirus genome and gene products in central nervous system tumours

**DOI:** 10.1038/sj.bjc.6602339

**Published:** 2005-02-08

**Authors:** J Sabatier, E Uro-Coste, I Pommepuy, F Labrousse, S Allart, M Trémoulet, M B Delisle, P Brousset

**Affiliations:** 1 Department of Neurosurgery, Purpan Hospital, Toulouse, France; 2 Department of Pathology, Rangueil Hospital, Toulouse, France; 3 Department of Pathology, University Hospital, Dupuytren, Limoges, France; 4 Department of Pathology, Purpan Hospital – INSERM U563 (CPTP), CHU Purpan, Toulouse, France

**Keywords:** central nervous system, glioma, cytomegalovirus, immunohistochemistry, *in situ* hybridisation

## Abstract

Human cytomegalovirus (HCMV) genome and related proteins have been reported in a great proportion of malignant gliomas. However, these results are unexpected since HCMV is not known as an oncogenic virus. By immunohistochemistry (with an anti-IE1 monoclonal antibody) and *in situ* hybridisation (with biotinylated DNA probes) on tissue microarrays and frozen sections, we investigated a French series of central nervous system (CNS) tumours, including 97 glioblastomas. In 10 cases of glioblastoma, rare astrocyte-like cells, admixed with tumour cells, stained positively for HCMV and in one case a doubtful staining of rare cells was noticed. This may indicate a reactivation of the virus under local immunosuppression but none of the cases of CNS tumours (*n*=132) contained HCMV genomes and/or proteins in a significant proportion of tumour cells. Our results strongly suggest that HCMV is unlikely to be implicated in the development of human malignant gliomas, at least in French cases.

A viral aetiology for human central nervous system (HCNS) tumours is provocative and remains a matter of strong debates. However, the discovery of a viral implication in CNS tumorigenesis may open different ways for preventing tumours with a constant fatal outcome such as glioblastomas. Accumulating data seem to indicate that SV40 is implicated in different tumours in humans and in particular in central nervous system (CNS) (Bergsagel *et al*, 1992; Klein *et al*, 2002; Carbone *et al*, 2003; Vilchez and Butel, 2003) However, there are strong controversies as to whether SV40 is directly linked to cancer development and a consensus is far from being reached in particular in lymphoma (Shivapurkar *et al*, 2002; Vilchez *et al*, 2002; Capello *et al*, 2003; MacKenzie *et al*, 2003).

A recent study has implicated the HCMV in the pathogenesis of malignant gliomas (Cobbs *et al*, 2002). HCMV is a *β*-herpesvirus able to infect various types of human cells, including glial cells (Britt and Alford, 1996). As other herpesviruses, HCMV infects 70–90% of the adult human population and persists in reservoir cells (Britt and Alford, 1996). Interestingly, in certain circumstances (immunosuppression), HCMV can be reactivated, thus undergoing a lytic cycle (Britt and Alford, 1996). However, except for non-Hodgkin's lymphoma, the incidence of primary CNS tumours is not increased in immunosuppressed patients. Moreover, despite recent investigations demonstrating the presence of HCMV in prostate and colorectal cancers (Harkins *et al*, 2002; Samanta *et al*, 2003), as yet this virus is not recognised as an oncogenic factor, at least *in vivo*.

In this study, we sought to detect HCMV infection in a series of French cases of CNS tumours by combining immunohistochemistry (IHC) and *in situ* hybridisation (ISH) on tissue microarrays.

## MATERIAL AND METHODS

### Tissues samples

Cases of CNS tumours were retrieved from our files at the Purpan Hospital in Toulouse between 1988 and 2003. Most cases were processed routinely, that is, fixed in Bouin's liquid and/or in 10% buffered formalin before paraffin embedding. These tumours consisted of 15 ependymomas, 81 glioblastomas and 20 oligodendrogliomas prepared in two blocks of tissue microarray (TMA) (Beecher Instruments, Micro-array technology, Sun Prairie, WI, USA). The age of the patients was between 31 and 87 years. Frozen material was available for nine patients with glioblastoma (among the 81 cases selected in tissue microarray). Six additional cases of glioblastoma from Toulouse (not included in the TMA) and 10 cases of glioblastoma from the University Hospital of Limoges were investigated because of the availability of frozen material. The approval of the French equivalent of the Institutional Review Board (Comité Consultatif de Préservation des Personnes en Recherche Biologique/CCPPRB) is not required for investigations based on archived paraffin and frozen blocks that have been routinely used for diagnostic purposes.

### Immunohistochemistry

**Figure 1:**
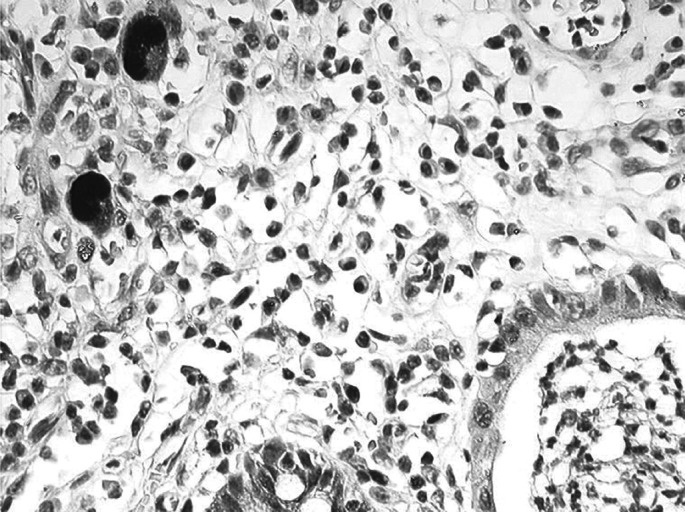
Immunodetection of HCMV with anti-IE1 (E13) antibody in a patient with acute colitis (peroxidase, × 500).

One anti-HCMV monoclonal antibody (clone E13, Argene-Biosoft, Varihes, France) was used in this study. It recognises an immediate early antigen of HCMV (IE1) and does not crossreact with EBV, Adenovirus, VZV and HSV. It works very well in positive controls (working dilution 1 : 100) and gives a strong nuclear staining in tissues with active HCMV infection (Figure 1). In addition, a weak cytoplasmic staining is seen in virtually all infected cells. In parallel, antigen retrieval and amplification with catalysed system amplification (CSA) (Dako, Carpintera, CA, USA) were used. This latter technique is on the order of 50-fold greater in sensitivity compared to standard IHC. The staining of the positive controls was obtained after standard antigen retrieval and/or amplification of the signal by CSA.

Immunostaining on paraffin sections was performed using the method described elsewhere with little modifications (Brousset *et al*, 2001). Briefly, paraffin sections were mounted on glass slides coated with silane (Sigma chemical Co., Saint Quentin, France). Sections were deparaffinised, placed in 10 mmol l^−1^ Na-citrate buffer (pH.6), and heated in a microwave oven (Whirlpool model; Philips, Eindhoven, Holland) at 900 W for cycles of 20 and 10 min. The slides were removed from the oven and allowed to cool for 30 min at room temperature. After washing in water, endogenous peroxidase was blocked with 1% hydrogen peroxide in methanol for 30 min. Slides were then rinsed in PBS before staining with a streptavidin–biotin three-stage technique, with the DAKO Strept ABC complex/HRP Duet kit (DAKO, code No. K492). For CSA technique, we used the kit from DAKO according to the supplier's recommendations. The DAKO CSA system HRP is an extremely sensitive immunohistochemical staining procedure incorporating a signal amplification method based on the peroxidase-catalysed deposition of a biotinylated compound followed by a secondary reaction with streptavidin peroxidase (DAKO). Immunostaining on frozen sections was performed according the alkaline phosphatase antialkaline phosphatase (APAAP) technique without prior antigen reactivation (Cordell *et al*, 1984). The working dilution of the antibody was identical to that of paraffin sections.

*Positive controls* consisted of lung biopsies (autopsy) of two HIV-positive patients and three colon biopsies (endoscopy) of nonimmunocompromised patients. Negative controls consisted of 10 normal (hyperplastic) lymph nodes and two cases of Hodgkin's disease. All these cases have been processed routinely, that is, fixed in Bouin's liquid and paraffin embedded. Frozen autopsy tissues of different organs from an immunocompromised patient (bone marrow transplantation) with generalised HCMV infection have also been included as positive controls.

In addition to anti-IE1 (E13) antibody, an anti-p52 ((nonstructural early DNA-binding protein (UL44 reading frame)) antibody (clone CCH2, Dako) was applied to frozen sections of the 10 additional cases from Limoges, with the same APAAP technique.

### *In situ* hybridization

This technique has been described elsewhere (Brousset *et al*, 1992). A biotinylated DNA probe from a commercial source (Enzo, PathoGene DNA probe assay, CMV DNA probe Pack, Part No 32802, Enzo life Sciences Inc., Faimingdale, NY, USA) was used according to the manufacterer's recommendations with some modifications (Brousset *et al*, 1992). In our hands, we found a correlation between ISH and IHC for control tissues.

## RESULTS

**Figure 2:**
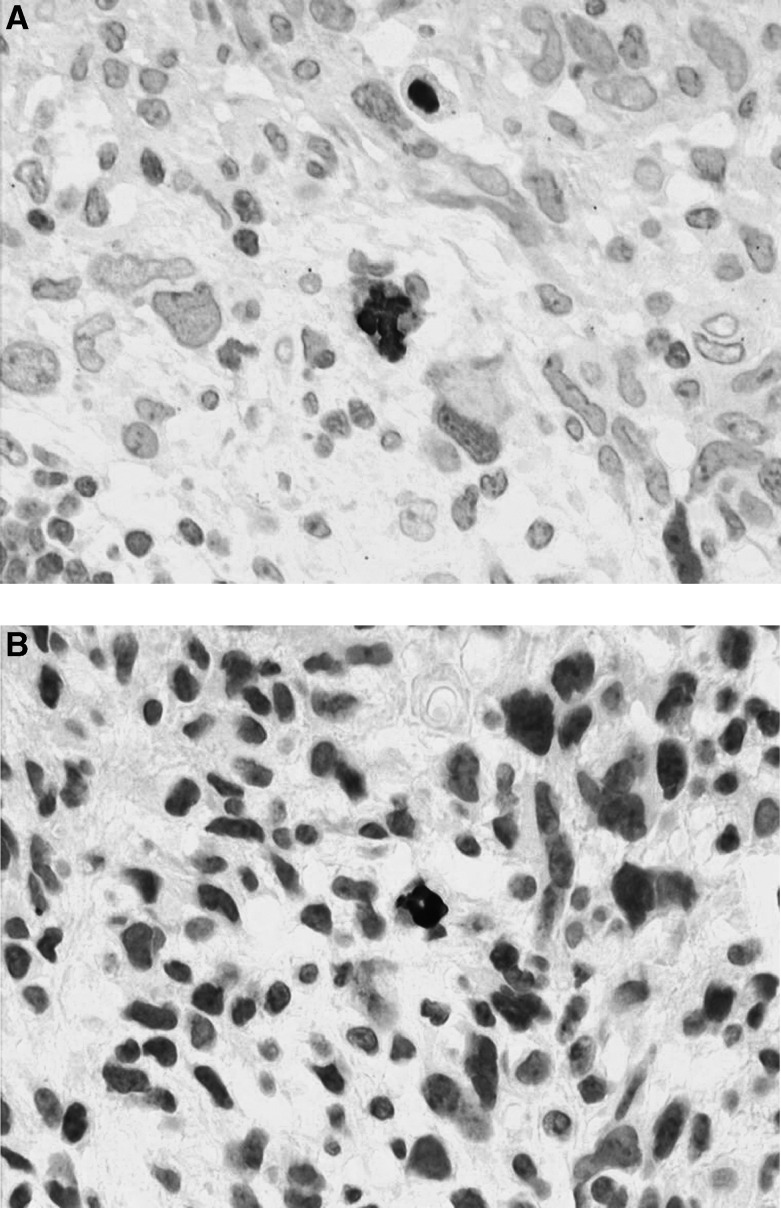
(**A**, **B**) Patterns of HCMV infection in two cases of glioblastoma. Note a strong positive nuclear and weak cytoplasmic staining with E13 Ab in isolated cells (peroxidase, × 800).

Human cytomegalovirus was detectable by IHC with or without amplification in all control tissues included in this study. The signal was strong, nuclear more or less cytoplasmic and was absent from all HCMV-negative tissues. There was no influence of the tissue processing on the results. We found a correlation between IHC and ISH for control tissues. In the series of 116 cases of CNS tumours, including 81 glioblastomas, we found HCMV-positive cells by IHC in only nine cases of glioblastoma, corresponding to isolated astrocyte like cells (one or two cells per slice). These cells were difficult to identify as reactive or neoplastic because of the possible modifications induced by viral infection (Figure 2A and B). No signs of endothelial cell infection were noticed. In these nine tumours and in all other tumours, we did not find HCMV infection in a significant proportion of cancer cells by both IHC (with and without amplification) and ISH. In nine cases of glioblastoma for which frozen material was available in Toulouse, we found scattered positive cells in one case only. This latter case had the same staining pattern on paraffin sections, whereas one case with positive cells on paraffin sections remained completely negative on frozen sections. The remaining cases were negative with both IHC methods. The six additional cases from Toulouse, not included in the TMA, were completely negative with anti-IE1. The 10 cases from Limoges were also negative with both anti-IE1 and anti-p52 antibodies despite a doubtful cytoplasmic staining of rare cells in one case.

Only seven out of the nine cases undoubtedly positive by IHC were detected by ISH (five different slices for all cases).

## DISCUSSION

The report by Cobbs *et al* (2002) is the first to show that HCMV nucleic acids and proteins are present in a high percentage of low- and high-grade malignant gliomas. In addition, they found the expression of early and delayed HCMV gene products in the tumours. The authors stated that their data did not establish a causal role for HCMV in glioma pathogenesis, but at least that HCMV could facilitate glioma progression through clonal expansion without producing a productive or cytopathic viral infection (Cobbs *et al*, 2002).

Our results roughly based on the same approach indicate that HCMV can be reactivated in scattered cells and in only a few cases of human gliomas. The presence of replicative viral genome and lytic proteins suggests that HCMV is reactivated, possibly under local immunosuppression. The site of its replication corresponds to *a priori* glial cells, the benign or malignant status of which could not be determined. These data are clearly at variance with those of Cobbs *et al* (2002). In addition, if HCMV had some role in CNS oncogenesis, one would expect to find the virus in all tumours cells as the result of a clonal expansion secondary to early infection by the virus. Our data do not support this hypothesis. In addition, an increased incidence of glial tumours and the description of HCMV-related tumours have not been reported in immunosuppressed patients.

The tissues we used in this study were mainly fixed in Bouin's liquid, which is well known to degrade nucleic acids (principally RNA). For these reasons, we did not use the RNA probe designed by Cobbs *et al* (2002). Indeed, most of mRNAs are degraded after Bouin's fixation. However, the use of a biotinylated DNA probe appears more suitable in this setting and has proven to be sensitive enough to detect HCMV genomes in cases with active viral replication (as suggested by IE1 expression). The latter idea was reinforced by the good correlation between IHC and ISH. However, the lack of perfect correlation between IHC and ISH can be easily explained by the scarcity of positive cells in each case (one or two per slices). One level in the block (one slice) can contain one or two positive cells but such cells could be absent in the next levels. The same explanation can be proposed to explain the discrepancy observed between fixed and frozen tissues.

Nevertheless, the most reliable and the simplest technique for detecting HCMV on fixed or frozen tissue sections is IHC with anti-IE1 antibodies. In their study, Cobbs *et al* (2002) have used this approach and we have confirmed that HCMV IE1 could be detected in some cases of glial tumours. However, contrary to the Cobbs *et al*'s (2002) study, the virus was mainly located in isolated cells suggesting a sporadic reactivation. The lack of IE1 detection in tumour cells on frozen sections is in keeping with the data obtained on paraffin sections. Interestingly, in 10 cases, the same results were obtained with the anti-p52 (CCH2) antibody. Since we used a reliable immunohistochemical technique (including signal amplification by CSA), our results can be compared with those of Cobbs *et al* (2002). We have no clue to understand the discrepancy of the results but epidemiological variations can be put forward to explain the difference of incidence of HCMV infection in glial tumours in France.

## References

[bib1] Bergsagel DJ, Finegold MJ, Butel JS, Kupsky WJ, Garcea RL (1992) DNA sequences similar to those of simian virus 40 in ependymomas and choroid plexus tumours of childhood. N Engl J Med 326: 988–993 1312224 10.1056/NEJM199204093261504

[bib2] Britt WJ, Alford CA (1996) Cytomegalovirus fields. In Fields Virology, Knipe BN, Howley DM, PM (eds) 3rd edn, pp 2493–2523. New York: Raven Press

[bib3] Brousset P, Butet V, Chittal S, Selves J, Delsol G (1992) Comparison of *in situ* hybridisation using different non isotopic probes for detection of Epstein–Barr virus in nasopharyngeal carcinoma and immunohistochemical correlation with anti-latent membrane protein antibody. Lab Invest 67: 457–464 1331609

[bib4] Brousset P, Cesarman E, Meggetto F, Lamant L, Delsol G (2001) Colocalization of the viral interleukin-6 with latent nuclear antigen-1 of human herpesvirus-8 in endothelial spindle cells of Kaposi's sarcoma and lymphoid cells of multicentric Castleman's disease. Hum Pathol 32: 95–100 11172301 10.1053/hupa.2001.21131

[bib5] Capello D, Rossi D, Gaudino G, Carbone A, Gaidano G (2003) Simian virus 40 infection in lymphoproliferative disorders. Lancet 361: 88–89 12517514 10.1016/S0140-6736(03)12157-5

[bib6] Carbone M, Pass HI, Miele L, Bocchetta M (2003) New developments about the association of SV40 with human mesothelioma. Oncogene 22: 5173–5180 12910254 10.1038/sj.onc.1206552

[bib7] Cobbs CS, Harkins L, Samanta M, Gillespie GY, Bharara S, King PH, Nabors LB, Cobbs CG, Britt WJ (2002) Human cytomegalovirus infection and expression in human malignant glioma. Cancer Res 62: 3347–3350 12067971

[bib8] Cordell JL, Falini B, Erber WN, Ghosh AK, Abdulaziz Z, MacDonald S, Pulford KA, Stein H, Mason DY (1984) Immunoenzymatic labeling of monoclonal antibodies using immune complexes of alkaline phosphatase and monoclonal anti-alkaline phosphatase (APAAP complexes). J Histochem Cytochem 32: 219–229 6198355 10.1177/32.2.6198355

[bib9] Harkins L, Volk AL, Samanta M, Mikolaenko I, Britt WJ, Bland KI, Cobbs CS (2002) Specific localisation of human cytomegalovirus nucleic acids and proteins in human colorectal cancer. Lancet 360: 1557–1563 12443594 10.1016/S0140-6736(02)11524-8

[bib10] Klein G, Powers A, Croce C (2002) Association of SV40 with human tumors. Oncogene 21: 1141–1149 11850833 10.1038/sj.onc.1205173

[bib11] MacKenzie J, Wilson KS, Perry J, Gallagher A, Jarrett RF (2003) Association between simian virus 40 DNA and lymphoma in the United Kingdom. J Natl Cancer Inst 95: 1001–1003 12837836 10.1093/jnci/95.13.1001

[bib12] Samanta M, Harkins L, Klemm K, Britt WJ, Cobbs CS (2003) High prevalence of human cytomegalovirus in prostatic intraepithelial neoplasia and prostatic carcinoma. J Urol 170: 998–1002 12913758 10.1097/01.ju.0000080263.46164.97

[bib13] Shivapurkar N, Harada K, Reddy J, Scheuermann RH, Xu Y, McKenna RW, Milchgrub S, Kroft SH, Feng Z, Gazdar AZ (2002) Presence of simian virus 40 DNA sequences in human lymphomas. Lancet 359: 851–852 11897287 10.1016/S0140-6736(02)07921-7

[bib14] Vilchez RA, Butel JS (2003) SV40 in human brain cancers and non-Hodgkin's lymphoma. Oncogene 22: 5164–5172 12910253 10.1038/sj.onc.1206547

[bib15] Vilchez RA, Madden CR, Kozinetz CA, Halvorson SJ, White ZS, Jorgensen JL, Finch CJ, Butel JS (2002) Association between simian virus 40 and non-Hodgkin lymphoma. Lancet 359: 817–823 11897278 10.1016/S0140-6736(02)07950-3

